# Novel pH-Sensitive Lipid Based Exo-Endocytosis Tracers Reveal Fast Intermixing of Synaptic Vesicle Pools

**DOI:** 10.3389/fncel.2018.00018

**Published:** 2018-02-02

**Authors:** Martin Kahms, Jürgen Klingauf

**Affiliations:** ^1^Department of Cellular Biophysics, Institute of Medical Physics and Biophysics, University of Münster, Münster, Germany; ^2^IZKF Münster and Cluster of Excellence Cells in Motion, University of Münster, Münster, Germany

**Keywords:** synaptic vesicle, exo-endocytosis, lipid tracer, resting pool, spontaneous activity

## Abstract

Styryl dyes and genetically encoded pH-sensitive fluorescent proteins like pHluorin are well-established tools for the optical analysis of synaptic vesicle (SV) recycling at presynaptic boutons. Here, we describe the development of a new class of fluorescent probes based on pH-sensitive organic dyes covalently bound to lipids, providing a promising complementary assay to genetically encoded fluorescent probes. These new optical tracers allow a pure read out of membrane turnover during synaptic activity and visualization of multiple rounds of stimulation-dependent SV recycling without genetic perturbation. Measuring the incorporation efficacy of different dye-labeled lipids into budding SVs, we did not observe an enrichment of lipids with affinity for liquid ordered membrane domains. But most importantly, we found no evidence for a static segregation of SVs into recycling and resting pools. A small but significant fraction of SVs that is reluctant to release during a first round of evoked activity can be exocytosed during a second bout of stimulation, showing fast intermixing of SV pools within seconds. Furthermore, we found that SVs recycling spontaneously have a higher chance to re-occupy release sites than SVs recycling during high-frequency evoked activity. In summary, our data provide strong evidence for a highly dynamic and use-dependent control of the fractions of releasable or resting SVs.

## Introduction

During synaptic transmission, neurotransmitters (NTs) stored in synaptic vesicles (SVs) are released by calcium-triggered exocytosis of membrane-docked vesicles. Subsequently, exocytosed synaptic membranes and proteins recycle by compensatory endocytosis (Ceccarelli et al., 1973; Heuser and Reese, 1973).

It has been proposed that SVs are organized in distinct SV pools. In hippocampal neurons, a readily releasable pool (RRP) comprises 10–15 docked SVs at the active zone of presynaptic boutons which can be depleted by 40–50 action potentials (APs) (Schikorski and Stevens, 2001). The reserve pool of SVs refills docking sites upon prolonged activity and together, RRP and reserve pool constitute the recycling pool of SVs, which can released upon AP-mediated activity. A further population of SVs is reluctant to release upon electrical stimulation and is classified as the resting pool [for review see (Denker and Rizzoli, 2010)].

However, in addition to evoked activity, synapses feature spontaneous release of NTs at resting intracellular calcium concentration with a frequency of about one vesicle per 90 s (Murthy and Stevens, 1999). The dynamics of both, the evoked and spontaneous exo-endocytic itinerary, have been studied by a combination of electrophysiology and fluorescence microscopy culminating in contradictory models with regard to the SV population driving spontaneous vs. evoked release (Prange and Murphy, 1999; Sara et al., 2005; Groemer and Klingauf, 2007; Fredj and Burrone, 2009; Hua et al., 2010; Wilhelm et al., 2010; Ramirez et al., 2012; Crawford et al., 2017).

In early studies, amphiphilic styryl dyes [FM-dyes (Betz and Bewick, 1992)] were utilized to visualize recycling of SVs that were selectively loaded with FM-dye either by evoked activity or during a period of silenced evoked activity by spontaneous turnover (Sara et al., 2005). These experiments suggested that SVs loaded with FM-dye during silenced evoked activity display reluctant release during subsequent evoked activity. Accordingly, SVs loaded with FM dye by evoked activity do not recycle during spontaneous turnover. These observations suggested that presynaptic boutons comprise distinct SV pools for evoked and spontaneous activity. However, these results were challenged by other publications reporting opposite results for comparable kind of experiments (Groemer and Klingauf, 2007; Wilhelm et al., 2010). Here, SVs recycling during a period of spontaneous activity displayed the same release probability upon subsequent evoked activity and *vice versa*. One possible explanation for this controversy might be difficulties in quantification and normalization of FM-release experiments as it was shown that a poor signal-to-noise ratio and erroneous normalization easily result in false kinetic profiles (Groemer and Klingauf, 2007).

Further studies employed different labeling strategies of SV proteins to clarify whether SVs recycling spontaneously and during activity belong to distinct SV pools. Experiments utilizing the endogenous reporter Synaptobrevin2-pHluorin, a pH-sensitive GFP mutant fused to the luminal domain of the vesicular SNARE-protein Synaptobrevin2 (Miesenbock et al., 1998), gave no evidence for a separation of SVs into distinct pools for evoked and spontaneous release (Hua et al., 2010; Wilhelm et al., 2010). Similar results were obtained when SV recycling was visualized by an exogenous labeling approach with fluorescently labeled antibodies directed against the luminal domain of the SV calcium sensor Synaptotagmin1 (Hua et al., 2010; Wilhelm et al., 2010). In contrast, selective enzymatic coupling of biotin to Synaptobrevin2 followed by exogenous labeling of recycling SVs with fluorescently labeled biotin again resulted in controversial results on the origin of spontaneously recycling SVs (Fredj and Burrone, 2009; Hua et al., 2010).

However, following different modes of SV recycling by tagging of SV proteins is an unbiased approach only under the assumption that all SVs share the same protein complement. Recently, it has been proposed that SVs carrying out spontaneous release utilize the non-canonical SNARE Vti1a and display a lower abundance of Synaptobrevin2 (Ramirez et al., 2012). In addition, asynchronously fusing SVs were found to be identified by the SNARE Vamp4 (Raingo et al., 2012). These findings culminated in a model of a heterogeneous pool of SVs with intrinsic differences in protein composition (Crawford and Kavalali, 2015).

Here, we describe new exogenous fluorescent probes based on the pH-sensitive organic cypHer5E dye coupled to phospholipids for optical analysis of presynaptic membrane trafficking independent of the respective protein complement. These optical tracers provide a promising complementary assay to genetically encoded fluorescent proteins and combine the appreciated features of both, FM styryl dyes and pHluorin fusion proteins. Like FM dyes, they do not rely on genetic modification and allow for labeling of only a subset of SVs, and like pHluorin-based sensors, they enable to monitor exo-endocytosis repeatedly at presynaptic sites. Using this novel class of optical tracers, we analyzed membrane turnover and SV re-use during several rounds of presynaptic activity and found no evidence for a static segregation of SVs into distinct SV pools for evoked and spontaneous activity. Instead, our results support a model of a use-dependent re-use of SVs.

## Materials and Methods

### Synthesis of Phospholipid-Fluorophore Conjugates

3 mg of 1,2-dimyristoyl-*sn-*glycero-3-phosphoethanolamine (DMPE), 1,2-dioleoyl-*sn*-glycero-3-phosphoethanolamine (DOPE) or *N*-lauroyl-D-*erythro*-sphingosyl-phospho-ethanolamine (Sphingo, all lipids purchased from Avanti Polar Lipids) were dissolved in dichlormethane/methanol (1:2) and added to a vial containing 1 mg cypHer5E-NHS (GE Healthcare). 10 μl of triethylamine were added and the mixture was stirred at room temperature for 1 h. Purification of the coupling product was performed by preparative thin layer chromatography (TLC) on silica plates (60 Å, layer thickness 1000 μm, Whatman) using a mixture of chloroform/methanol/water (65:25:4) as developing solvent. The product was extracted with chloroform/acetonitril/methanol (1:1:1). Solvent was evaporated and the conjugate was dissolved in DMSO. Concentration was determined after dilution in carbonate buffer pH 8.5 by spectroscopic analysis assuming an extinction coefficient of 40.000 M^-1^cm^-1^ at 500 nm. Coupling products were characterized by ESI-MS mass spectrometry and stored at concentrations from 500 to 1000 μM under argon atmosphere at -20°C.

### Plasmids

The plasmid encoding Syb2-pHl has been described before (Wienisch and Klingauf, 2006). The plasmid encoding for Synaptophysin1-pHluorin was a gift from L. Lagnado (MRC, Cambridge, United Kingdom).

### Cell Culture

Dissociated cultures of mouse hippocampal neurons were prepared from CA3/CA1 regions of 1 to 3-day-old CD1 mice of either sex as described previously (Liu and Tsien, 1995). All animals were treated in accordance with the regulations and guidelines of the State of North Rhine-Westphalia. Transfection was performed at day 4 *in vitro* (DIV) by a modified calcium phosphate transfection procedure (Threadgill et al., 1997). Experiments were carried out at DIV 15–25.

### Epifluorescence Microscopy

All experiments, unless otherwise stated, were carried out in 140 mM NaCl, 2.4 mM KCl, 2.5 mM CaCl_2_, 1.3 mM MgCl_2_, 10 mM glucose, 10 mM HEPES, pH 7.4. NH_4_Cl solution was prepared by substituting 50 mM NaCl in standard buffer with NH_4_Cl, calcium-free buffer was prepared by substituting CaCl_2_ with MgCl_2_. For lipid staining, stock solutions in DMSO were diluted to a final concentration of 1 μM (<0.2% DMSO) and rigorously vortexed. After incubation of cells with the lipid suspension for 2 min at room temperature, cells were washed twice with standard buffer.

Imaging was performed at room temperature on an inverted Zeiss 100 microscope equipped with a 63×, 1.2 NA water-immersion objective. Images were acquired with a Neo sCMOS camera (Andor) controlled by IQ software (Andor) in 2 × 2 binning mode. CypHer5E was excited at 640 nm, pHluorin and FM 1–43 at 480 nm with a computer-controlled monochromator (Polychrome V, Till Photonics) and fluorescence was detected after passing a FITC/Cy5 dual-band filter set (AHF Analysentechnik). Typically, time-lapse images were acquired at 0.5 Hz. For dual-color recordings, alternating images in the green and red channel were acquired at 0.5 Hz. Neurons were stimulated by electric field stimulation (platinum electrodes, 10 mm spacing, 1 ms pulses of 50 mA and alternating polarity at 5–50 Hz) applied by constant current stimulus isolator (WPI A 385, World Precision Instruments). 10 μM 6-cyano-7-nitroquinoxaline-2,3-dione (CNQX) and 50 μM D,L-2-amino-5-phosphonovaleric acid (AP5) were added to prevent recurrent activity. Solution exchange was achieved through a glass tubing perfusion system controlled by a piezo-controlled stepper device (SF778, Warner Instruments).

### Data Analysis

Quantitative analysis was performed with self-written macros in Igor Pro (Wavemetrics). To avoid bias introduced by manual selection of functional boutons, an automated detection algorithm was used. The image from the time series showing maximum response during stimulation was subjected to an á trous wavelet transformation as described previously (Wienisch and Klingauf, 2006). All identified masks and calculated time courses were visually inspected for correspondence to individual functional boutons.

### Fluorescence Correlation Spectroscopy (FCS)

Fluorescence correlation spectroscopy (FCS) measurements were performed on a Leica TCS 4Pi microscope (Leica Microsystems) of type A running in the confocal mode with an instrumental upgrade for FCS applications (Vista FCS, ISS, Champaign, IL, United States). The microscope was equipped with a 100×/1.46 N.A. oil immersion objective. DMPE-CypHer5E fluorescence was excited by 633 nm light of a HeNe-laser and emitted light was collected by a photon counting avalanche photodiode (Perkin Elmer, Foster City, CA, United States) after passing a HQ665LP filter (Chroma Technologies). The detection pinhole was set to 1 Airy unit. Correlation was performed in time mode with a sampling frequency of 50 kHz. Autocorrelation curves (ACs) were recorded on stained plasma membrane patches of somata of immature neurons (DIV 4–6) at pH 5.5 to dequench the majority of lipids resident at the plasma membrane and avoid uptake of fluorescent lipid into SVs at mature synapses. ACs were fitted to the following equation considering 2D-diffusion and proton equilibrium:

$$\begin{array}{c} G(\tau )=\frac{\left[1-P+P*\exp\left(-\frac{\tau }{\tau_{\text{c}}}\right)\right]}{N*(1-P)}*{\left(1+\frac{\tau }{\tau_{\text{D}}}\right)}^{-1} \end{array}$$

with

P: chemically induced dark fraction

τ_C_: chemical relaxation time

N: number of molecules in confocal volume

τ_D:_ diffusion time of molecular species

The diffusion time is related to the confocal volume by

with

$$\begin{array}{c} \tau_{\text{D}}=\frac{r^{2}}{4D} \end{array}$$

D: diffusion coefficient

r: 1/e^2^ decay of laser intensity in lateral direction

*r* was determined to 289 ± 19 nm by imaging fluorescent beads of sub-resolution 40 nm size. A chemically induced dark fraction was introduced to account for an initial fast decay of the AC with a half-life of ∼350 μs, which can be most likely attributed to protonation/deprotonation dynamics (Haupts et al., 1998).

## Results

### DMPE-cypHer5E Stains Synaptic Vesicles in Hippocampal Neurons

**FIGURE 1:**
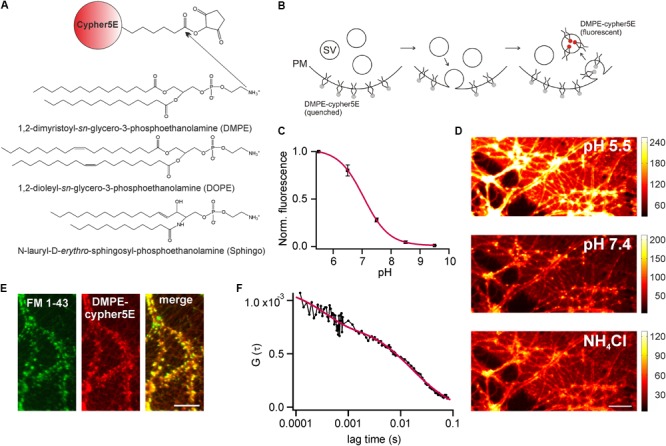
DMPE-cypHer5E stains synaptic vesicles (SVs) in primary hippocampal neurons. **(A)** Principle of lipid-dye coupling: The NHS-ester of cypHer5E was coupled to the primary amine of different lipids in organic phase. Unreacted dye was removed by thin-layer chromatography. **(B)** Schematic illustration depicting fluorescence changes of DMPE-cypHer5E during SV recycling. PM, plasma membrane; SV, synaptic vesicle. **(C)** Average normalized membrane fluorescence of hippocampal neurons stained with DMPE-cypHer5E as a function of pH (*n* = 3 coverslips, 10 ROIs each). Solid line represents a fit to a Henderson–Hasselbalch equation resulting in a pK of 7.1. **(D)** Neurons stained with pH-sensitive DMPE-cypHer5E displayed a highly fluorescent plasma membrane at acidic pH. After loading of SVs with DMPE-cypher5E (20 min network activity at 37°C and subsequent stimulation with 900 APs at 20 Hz), imaging at pH 7.4 revealed a punctate pattern of fluorescence that was quenched by superfusion with NH_4_Cl. Scale bar: 10 μm. **(E)** Co-localization of FM 1-43 and DMPE-cypHer5E fluorescence signals. Scale bar: 10 μm. **(F)** Average autocorrelation curves (*n* = 6) of DMPE-cypHer5E in the plasma membrane of immature neuronal cultures at pH 5.5. Solid red line represents fit to a 2D-diffusion model with an additional term accounting for the fast decay at low correlation times (*N* [number of molecules in confocal volume] = 1189, τ_D_ [diffusion time] = 16.4 ms, τ_c_ [chemical relaxation time] = 350 μs, P [chemically induced dark fraction] = 0.29). The estimated diffusion time corresponds to a diffusion coefficient of 1.3 μm^2^ s^-1^, in good agreement with earlier estimates for phospholipid mobility in neuronal membranes (Renner et al., 2009). Error bars represent SEM.

We established optical probes for the analysis of membrane trafficking in single synaptic boutons by coupling the NHS-ester of the cypHer5E-fluorophore (Adie et al., 2002) to the primary amine of different phospho- and sphingolipids (**Figure 1A**). The cypHer5E dye exhibits maximal fluorescence at acidic pH of around 5 and is quenched at neutral to alkaline pH, thus providing a pH-dependency applicable to sense pH-changes associated with SV recycling (**Figure 1B**). We first conjugated the short-chain saturated phospholipid 1,2-dimyristoyl*-sn*-glycero-3-phosphoethanolamine (DMPE, **Figure 1A**) to the cypHer5E dye and found that the outer leaflet of the plasma membrane of primary hippocampal neurons could be readily stained by the lipid-dye conjugate with maximum membrane fluorescence at an extracellular pH of 5 (**Figures 1C,D**). The fluorescent signal was quenched by almost one order of magnitude at alkaline pH with a pK value of 7.1 for membrane bound lipid-dye conjugate (**Figure 1C**), providing a sufficient dynamic range to measure pH-changes during SV recycling.

Next, we stained primary hippocampal neurons with DMPE-cypher5E, incubated them for 20 min at 37°C (to allow incorporation of lipid-dye conjugate into SVs by endogenous network activity) and challenged them by 900 APs to mobilize the entire recycling pool. After stimulation, a punctate fluorescence pattern became visible which disappeared upon superfusion with NH_4_Cl (**Figure 1D**). Ammonium-sensitivity of the fluorescent puncta suggested incorporation of DMPE-cypHer5E into acidified SVs. This assumption was verified by dual-color imaging of lipid fluorescence and the fluorescent signal of the styryl dye FM 1–43 (Betz and Bewick, 1992). DMPE-cypHer5E-stained neurons were challenged by a loading stimulus in presence of 5 μM FM 1–43 (900 APs at 20 Hz). After styryl dye washout, we observed a strong co-localization of DMPE-cypHer5E and FM 1–43 signals (**Figure 1E**).

We performed fluorescence correlation spectroscopy (FCS) on somata of stained immature neurons (DIV 5) to estimate the number of lipid molecules per unit membrane area (**Figure 1F**). Analysis of autocorrelation curves revealed an average number of ∼4500 DMPE-cypHer5E molecules per μm^2^. Assuming a homogeneous distribution of the fluorescent lipid for plasma and vesicular membranes, this value corresponds to ∼ 20 DMPE-cypHer5E molecules per SV, indicating a low ratio of introduced fluorescent reporter to endogenous lipids (Takamori et al., 2006).

### Monitoring Lipid Recycling in Individual Hippocampal Boutons

**FIGURE 2:**
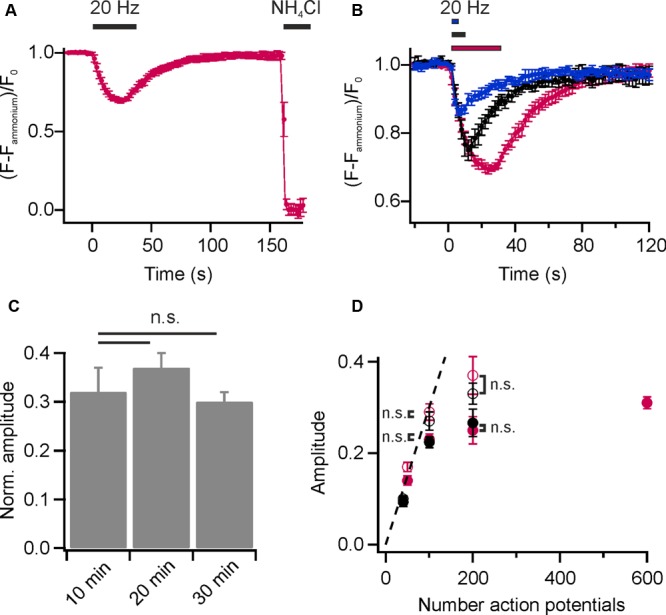
Monitoring lipid recycling in individual hippocampal boutons. **(A)** Average normalized fluorescence signal of presynaptic boutons loaded with DMPE-cypHer5E (20 min network activity at 37°C and subsequent stimulation with 900 APs at 20 Hz) in response to a stimulation train of 600 APs at 20 Hz and NH_4_Cl-superfusion. The response amplitude was normalized to the total internalized DMPE-cypHer5E fraction uncovered by superfusion with NH_4_Cl. (*n* = 5 coverslips, 182 to 273 boutons each). **(B)** Average normalized fluorescence signals of synaptic boutons loaded with DMPE-cypHer5E in response to different numbers of APs at 20 Hz (blue: 50 APs, black: 200 APs, red: 600 APs; *n* = 5 coverslips, 84 to 394 boutons each). Traces were normalized to the fluorescence signal during NH_4_Cl-superfusion. **(C)** Release amplitudes (200 APs, 20 Hz) normalized to the fluorescence signal during NH_4_Cl-superfusion are similar for experiments performed 10, 20 and 30 after loading of SVs with DMPE-cypher5E (*n* = 4 coverslips, 44 to 255 boutons each; n.s., not significant, *p* > 0.4, unpaired *t*-test). **(D)** Apparent exocytosis amplitudes (red, filled circles) plotted as a function of AP number together with amplitudes corrected for endocytosis during stimulation by back-extrapolation (red, open circles). For comparison, the same values are plotted for the endogenous tracer Syb2-pHluorin (black, filled circles: apparent exocytosis amplitudes; black, open circles: amplitudes corrected by back-extrapolation; n.s., not significant, *p* >0.8, unpaired *t*-test). All error bars represent SEM.

To visualize presynaptic activity at individual boutons, we again loaded the recycling pool of SVs with DMPE-cypHer5E (20 min endogenous network activity at 37°C and subsequent stimulation with 900 APs at 20 Hz) and performed time-lapse imaging. Electrical field stimulation at 20 Hz caused a rapid decay in cypHer5E-fluorescence at individual boutons followed by a slow exponential recovery of fluorescence reporting the pH-changes accompanied with exocytosis and reacidification after endocytosis of SVs (**Figure 2A**). For amplitude normalization, we applied an ammonium pulse at the end of the experiment to equilibrate the pH across all membranes and unmask the total internalized cypHer5E fraction (**Figure 2A**). Normalized amplitudes scaled with increasing stimulus strength (**Figure 2B**), and we estimated the time constant of fluorescence recovery for 200 APs at 20 Hz by mono-exponential fits, resulting in τ = 19.1 ± 0.95 s. This value is in good agreement with pHluorin-based measurements (Bodzeta et al., 2017). We found no difference in NH_4_Cl-normalized release amplitudes for experiments performed 10, 20, or 30 min after initial loading of SVs with DMPE-cypher5E (**Figure 2C**). We concluded that normalization of release amplitudes to the signal upon NH_4_Cl superfusion is robust and not significantly weakened by additional lipid uptake due to housekeeping endocytosis or enrichment in endosomes and other trafficking organelles. Previous studies have shown that the initial rate of endocytosis after stimulus is similar to the endocytic rate during the stimulus period (Sankaranarayanan and Ryan, 2000; Fernandez-Alfonso and Ryan, 2004). Thus, we could estimate the pure exocytosis amplitude by back-extrapolating a linear fit to the initial post-stimulus rate of endocytosis. Back-extrapolated amplitudes scaled linearly with increasing stimulus strength up to 100 APs, comparable to release amplitudes obtained for the endogenous tracer Synaptobrevin2-pHluorin (Syb2-pHl; **Figure 2D**).

In addition, this approach is readily applicable to study SV recycling in acute preparations of non-culturable cells like bipolar cells of the retina, where application of genetically encoded probes was not possible to date (Supplementary Figures S1A–C).

Taken together, these results show that DMPE-cypHer5E is a reliable reporter of lipid recycling during synaptic activity and does not impair exo-endocytic cycling, unlike long-term incubation with high concentrations of exogenous phospholipids (Uchiyama et al., 2007).

### Comparison of DMPE-cypHer5E and Established Optical Reporters

**FIGURE 3:**
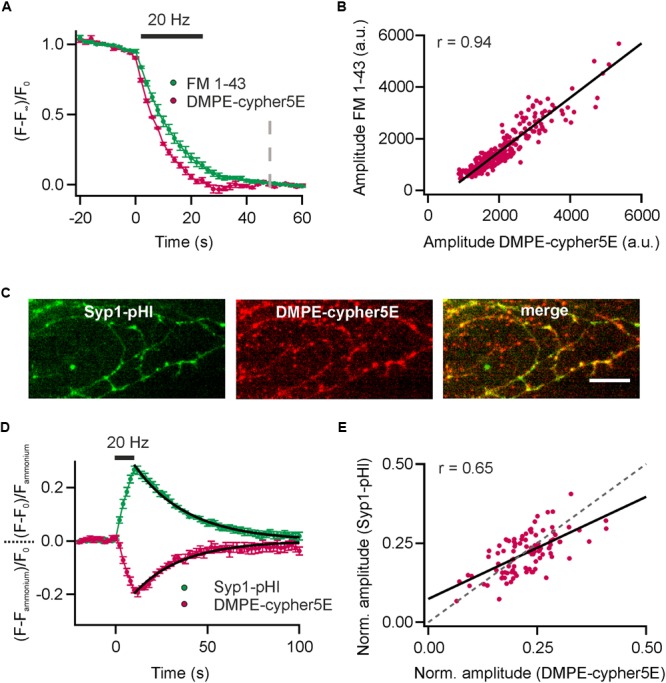
Comparison of DMPE-cypHer5E and established optical reporters. **(A)** Dual-color analysis of FM 1–43 destaining and exocytosis registered by decrease in DMPE-cypHer5E signal in presence of 65 nM Folimycin for a stimulation train 600 APs at 20 Hz. Apparent time constants of exocytosis differed significantly indicating slow clearance of FM 1-43 out of the synaptic cleft. (*n* = 4 coverslips; 120 to 354 boutons each). Dashed line: time point of amplitude quantification. **(B)** Absolute amplitudes (i.e., camera counts) of FM 1–43 and DMPE-cypHer5E responses extracted from the same boutons showed strong correlation (plotted for a single experiment, *n* = 280 boutons; solid line represents linear fit). **(C)** Fluorescence signals of Syp1-pHl (maximal peak fluorescence upon a train of 200 APs at 20 Hz) and DMPE-cypher5E (maximal fluorescence during baseline acquisition) display partial co-localization of individual boutons. Note that our transfection method targets only 10–20% of neurons resulting in a significant fraction of of DMPE-cypher5E-positive boutons that do not express Syp1-pHl. Scale bar: 10 μm. **(D)** Average fluorescent signals for both, Syp1-pHl and DMPE-cypHer5E, extracted from double-labeled boutons normalized to the NH_4_Cl-responses for a stimulation train of 200 APs at 20 Hz (*n* = 5 coverslips, 47 to 135 boutons each). Both averaged traces showed near identical time courses of endocytosis as estimated by mono-exponential fits (black line, τ_DMPE_ - -_cypHer5E_ = 26 s, τ_Syp1-pHl_ = 29 s). **(E)** NH_4_Cl-normalized amplitudes of Syp1-pHl and DMPE-cypHer5E responses extracted from the same boutons showed reasonable correlation (plotted for a single experiment, *n* = 92 boutons; solid line represents linear fit, dashed line corresponds to unity line of slope 1). r, Pearson correlation coefficient. All error bars represent SEM.

Since cypHer5E fluoresces in the red spectrum (maximum excitation around 640 nm), measurements of lipid recycling can be easily combined with optical probes emitting at shorter wavelength. We performed dual-color recordings of FM 1–43 release and exocytosis registered by loss of DMPE-cypHer5E fluorescence for a stimulus train of 600 APs at 20 Hz in presence of the v-ATPase blocker Folimycin at the same synaptic boutons. Using this so-called alkaline trapping method (Li et al., 2005), we separated the exocytosis kinetics in the cypHer5E-channel from those of endocytosis resulting in pure time traces of exocytosis for both recording channels. By applying the mask of reactive boutons in the green channel (FM 1–43) to the time-lapse images in the red channel, we extracted the DMPE-cypHer5E signals at the same boutons (**Figure 3A**). Analysis revealed a strong correlation of absolute release amplitudes for both exogenous optical tracers (**Figure 3B**). However, in case of FM 1–43, a reliable normalization of release amplitudes to the total internalized dye is not possible (because of undefined background). Therefore, we could only correlate absolute release amplitudes (i.e., camera counts) of both detection channels. As expected, time constants of fluorescence decay differed significantly with Δτ_1/2_ of about 3 s, in line with earlier indirect estimates of FM-dye clearance out of the synaptic cleft (Ryan et al., 1996; Klingauf et al., 1998; Richards et al., 2000). Note that clearance out of the synaptic cleft is much slower than departitioning out of an exposed membrane (Wu et al., 2009).

Dual-color recordings of Synaptophysin1-pHluorin (Syp1-pHl) transfected neurons stained with DMPE-cypHer5E (**Figure 3C**) displayed kinetic transients of DMPE-cypHer5E that mirror the Syp1-pHl signal with respect to both exocytic and endocytic kinetics (τ_decay_ ∼26 s vs. ∼29 s obtained by mono-exponential fits, **Figure 3D**). Release amplitudes for Syp1-pHl and DMPE-cypHer5E normalized to the NH_4_Cl response showed a weaker correlation compared to release amplitudes for FM 1–43 and DMPE-cypHer5E (**Figure 3E**). This finding might be explained by the fact that in contrast to DMPE-cypher5E, endogenously expressed Syp1-pHl labels not only the recycling fraction of SVs but the complete SV pool. However, the significant correlation argues that on the timescale of our experiments (staining and network activity for 20 min, followed by 900 APs at 20 Hz and 5 min rest before experimental recording) normalization to the NH_4_-signal yields a robust estimate of fractional release. Note that due to a low transfection efficiency not all DMPE-cypher5E labeled boutons express Syp1-pHl (**Figure 3C**). However, we found similar exo-endocytosis kinetics measured with DMPE-cypher5E for boutons colocalizing with Syp-pHl and boutons that do not colocalize with Syp1-pHl (Supplementary Figures 2A–C).

It has been reported that some FM-dyes exhibit surfactant-like properties and perturb the mechanical properties of membranes already at low μM concentrations As a result of this perturbation, the release probability of SVs is decreased (Zhu and Stevens, 2008). To test whether DMPE-cypher5E exhibits similar properties, we compared Syp1-pHl responses of DMPE-stained neurons to the responses of non-stained neurons for a stimulus train of 200 APs at 20 Hz. We could not detect any difference in amplitudes (normalized to ammonium) or post-stimulus decay kinetics (Supplementary Figures S3A,B) and concluded that the mechanical properties of neuronal membranes are not affected for the DMPE-cypher5E concentrations we used.

### DOPE-cypHer5E Displays Higher Incorporation Efficacy into Budding SVs Compared to Lipids with Affinity for Liquid Ordered Lipid Phases

We prepared two additional probes and coupled cypHer5E to 1,2-oleyl*-sn*-glycero-3-phosphoethanolamine (DOPE) and *N*-lauroyl-D-*erythro*-sphingosyl-phospho-ethanolamine (Sphingo, **Figure 1A**). The unsaturated oleyl-moieties of DOPE are the most abundant fatty acids in brain lipid extracts (Biran and Bartley, 1961) and are excluded from liquid ordered lipid phases (Schutz et al., 2000). Sphingolipids in contrast, are transiently trapped in such lipid nanodomains (Eggeling et al., 2009).

**FIGURE 4:**
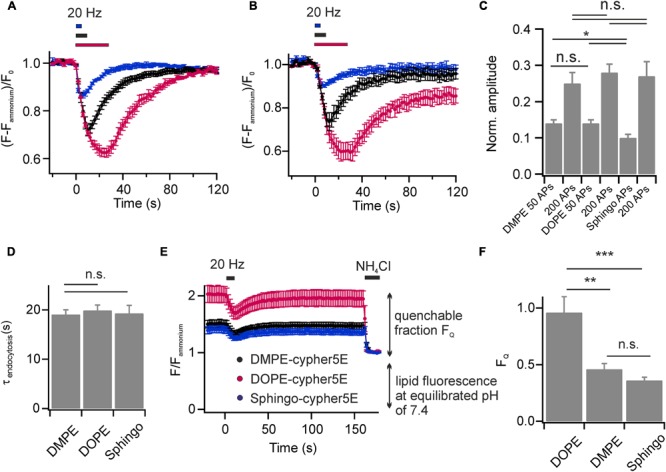
Lipids with affinity for liquid ordered membrane domains are not enriched in budding SVs. Average normalized fluorescence signals of boutons stained with **(A)** DOPE-cypHer5E and **(B)** Sphingo-cypHer5E in response to trains of APs at 20 Hz (blue: 50 APs, black: 200 APs, red: 600 APs; *n* = 5 coverslips, 54 to 398 boutons each). Traces were normalized to the NH_4_Cl responses. **(C)** Apparent exocytosis amplitudes for the three lipid tracers in response to 50 and 200 APs. **(D)** Time constants of fluorescence recovery for a stimulation train of 200 APs at 20 Hz (estimated by mono-exponential fits) for the three lipid tracers n.s., not significant, *p* > 0.6, unpaired *t-*test). **(E)** Quantification of lipid incorporation into budding SVs. Neurons were challenged by a test pulse (200 APs at 20 Hz) to identify functional boutons. Fluorescence decay amplitudes in presence of NH_4_Cl at individual boutons relative to the remaining fluorescence at an equilibrated pH of 7.4 provided an estimate of the amount of cypHer5E labeled lipids incorporated into acidified vesicles (black: DMPE, red: DOPE, blue: Sphingo; *n* = 7 coverslips, 85 to 398 boutons each). Traces were normalized to background fluorescence and total lipid fluorescence at an equilibrated pH of 7.4. **(F)** Quantification of traces shown in **(E)** revealed highest incorporation efficacy for the unsaturated lipid DOPE-cypHer5E (^∗^*p* < 0.02, ^∗∗^*p* < 0.01, ^∗∗∗^*p* < 0.002; n.s., not significant, *p* > 0.17, unpaired *t*-test). All error bars represent SEM.

We found that DOPE- and Sphingo-cypHer5E both labeled the plasma membrane of hippocampal neurons and were incorporated into SVs upon endocytosis. Stimulation induced fluorescent transients were comparable to those obtained for DMPE-cypHer5E (**Figures 4A,B**) in terms of release amplitudes (**Figure 4C**) and time constants of fluorescence recovery (**Figure 4D**). Notably, for Sphingo-cypher5E we observed a slightly reduced release amplitude for 50 APs at 20 Hz and a tendency of incomplete fluorescence recovery for intense stimulation paradigms. These findings might reflect a more complex sorting of sphingolipids into budding SVs like, e.g., partial exclusion from SVs when retrieval is driven by bulk endocytosis (Clayton and Cousin, 2009).

We next sought to estimate the relative incorporation efficacy of the lipid probes into budding SVs. First, we applied a test stimulus (200 APs, 20 Hz) to identify functional boutons (**Figure 4E**). Then, we determined fluorescence responses upon NH_4_Cl superfusion at identified responding boutons relative to the total membrane fluorescence at an equilibrated pH of 7.4. The highest NH_4_Cl-quenchable fraction *F*_Q_, and therefore highest incorporation efficacy, was found for the unsaturated phospholipid DOPE-cypHer5E, while DMPE-cypHer5E and Sphingo-cypHer5E were less efficiently incorporated into SVs (**Figure 4F**). These data are consistent with the notion that the SV membrane has no obvious raft-like character (Takamori et al., 2006). Instead, lipid incorporation into SVs might be primarily curvature-driven as it has been shown that both, DMPE and DOPE insert preferentially into the inner leaflet of small artificial vesicles *in vitro*, but that DOPE displays an even higher preference for highly curved membranes (Kamal et al., 2009). The differences in incorporation efficiency we observed for DMPE/Sphingo and DOPE somewhat question the assumption of our FCS analysis (**Figure 1F**), that fluorescent lipid concentrations are identical in plasma and vesicular membranes. Since, however, concentrations differ by far less than one order of magnitude our FCS-analysis on somatic membranes provides a reasonable estimate of the number of molecules per SV.

### Uptake of Exogenous cypHer5E-Labeled Lipids into Recycling SVs Reveals a Dynamic SV Pool Organization

**FIGURE 5:**
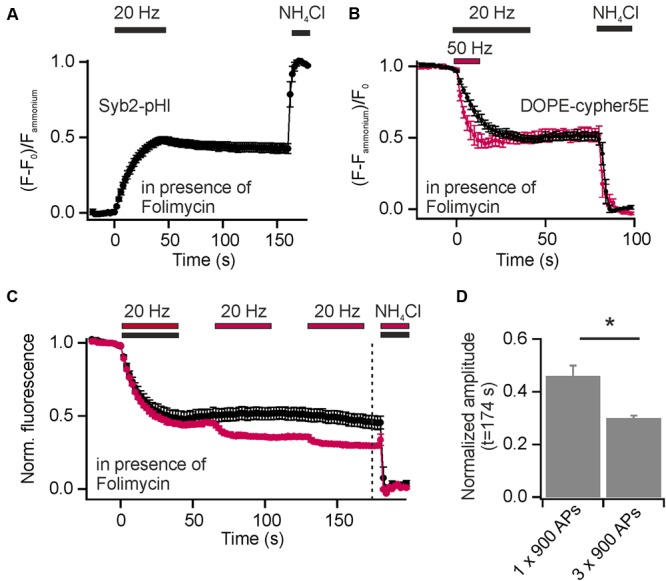
Incorporation of exogenous DOPE-cypHer5E into SVs reveals a highly dynamic SV pool organization. **(A)** Average normalized cumulative release for Syb2-pHl in response to a depleting stimulus (900 APs, 20 Hz) in presence of 65 nM Folimycin (*n* = 7 coverslips, 50 to 237 boutons each). **(B)** Average normalized cumulative releases (decay of DOPE-cypHer5E fluorescence in presence of Folimycin normalized to NH_4_Cl response) for two depleting stimulus trains [600 APs at 50 Hz (red) and 900 APs at 20 Hz (black)]. About 50% of SVs labeled with the exogenous tracer can be released during a single stimulus train at 20 or 50 Hz (*n* = 5 coverslips, 143 to 343 boutons each). **(C)** Average normalized cumulative releases like in **(B)**, but for three 65 s spaced stimulus trains (red; 3x 900 APs at 20 Hz; *n* = 4 coverslips, 110 to 291 boutons each) together with control measurements of same recording time but only one stimulus train (black; 1x 900 APs at 20 Hz; *n* = 5 coverslips, between 111 and 367 boutons each; dashed line: time point of amplitude quantification). **(D)** Quantification of release amplitudes for the experiment shown in **(C)** shows slow but consistent mixing of reluctantly releasable SVs with the recycling pool (^∗^*p* < 0.02), unpaired *t*-test. All error bars represent SEM.

Synaptic vesicles are organized in distinct SV pools: a recycling pool (comprising docked SVs at the active zone and SVs that refill docking sites), and the resting pool, a population of SVs reluctant to release upon electrical stimulation. The fractional size of the recycling pool of SVs can be quantified using Syb2-pHl transfected neurons and applying a depleting stimulus (900 APs at 20 Hz) in presence of Folimycin. By subsequent perfusion with NH_4_Cl, the maximal cumulative release can be determined (**Figure 5A**). With this experiment, we found equal amounts of SVs in the recycling and the reluctantly releasable resting pool for presynaptic boutons of hippocampal neurons, as already shown previously (Hua et al., 2010).

In contrast to endogenous pHluorin-based reporters, which tag all SVs in the presynapse, the exogenous cypHer5E-based lipid tracers label selectively SVs, which underwent at least one cycle of exo-endocytosis after membrane staining. However, release amplitudes, normalized to the total pool of labeled SVs, saturate with increasing stimulus strength (**Figures 2A,B**). Furthermore, normalized release fractions for both, exogenous DMPE-cypHer5E and endogenous Syp1-pHl, show significant correlation (**Figure 3E**), pointing to a population of SVs that took up the exogenous tracer in a first round of stimulation but is locked during a further bout of evoked activity.

Inspired by these observations we started analysis of SV pool dynamics using DOPE-cypHer5E, being the tracer most efficiently incorporated into SVs. First, we quantified the apparently release-reluctant SV fraction in presence of Folimycin and found, that 5 min after loading of SVs with DOPE-cypHer5E (900 APs at 5 Hz to avoid bulk endocytosis) delivery of depleting stimuli (600 APs at 50 Hz or 900 APs at 20 Hz) mobilized only about 50% of stained SVs (**Figure 5B**). This value is in good agreement with data obtained for Syb2-pHl (**Figure 5A**), but for an exogenous tracer like DOPE-cypHer5E this finding suggests a conversion of SVs from the recycling pool to the resting pool and thus, mixing of these pools on a timescale of a few minutes. Indeed, the cumulative release amplitude dropped to ∼30% after three 65 s spaced stimulus trains (900 APs at 20 Hz), which is an increase in signal decay by ∼15% compared to control measurements with only one stimulus train but same recording time (**Figures 5C,D**). These data show that at least one third of the apparent resting pool can be mobilized during further bouts of stimulation.

We routinely performed our measurements on DIV 15–25. To test if intermixing of SV pools depends on the maturity of the synaptic contacts, we repeated the latter experiment on cultures at DIV 11–12 (Supplementary Figure 4A). Quantification revealed only a minor reduction in release amplitudes, which is hardly significant (Supplementary Figure 4B). However, the distribution of release amplitudes for single boutons is much broader for younger neurons (Supplementary Figure 4C), indicating that boutons converge to similar SV pool organization with increasing age. Furthermore, the cumulative release amplitudes for the first and second stimulus train are not correlated for individual boutons, i.e., boutons that are less destained during the first round of stimulation do not display stronger destaining during the next round or vice versa (Supplementary Figure 4D).

**FIGURE 6:**
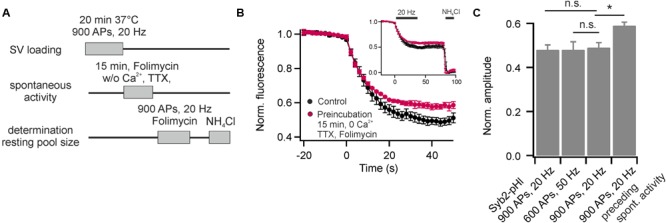
The recycling pool of SVs is the source of spontaneous release. **(A)** Experimental paradigm to probe the origin of spontaneously recycling SVs. **(B)** Recycling pool size probed with 900 APs at 20 Hz in presence of Folimycin for control (black) and after pre-incubation of neurons for 15 min at 37°C in presence of TTX, zero external calcium and Folimycin (red). The inset shows complete traces including NH_4_Cl-superfusion (*n* = 5 coverslips, 150 to 350 boutons each). **(C)** Quantification of the recycling pool size for Syb2-pHl (shown in **Figure 5A**) and for DOPE-cypher5E [shown in **Figure 5B** and **(B)**]. The reduction in apparent recycling pool size after a period of spontaneous activity in presence of Folimycin indicates a cross-depletion of the recycling pool by preceding spontaneous activity. Thus, most spontaneously recycled SVs were drawn from the recycling pool. (^∗^*p* < 0.02; n.s., not significant, *p* > 0.4, unpaired *t*-test). All error bars represent SEM.

It has been speculated that the resting pool of SVs might constitute the source for SVs that fuse spontaneously, i.e., fuse independent of AP-mediated calcium influx (Fredj and Burrone, 2009). Therefore, we tested the apparent size of the recycling pool after preceding spontaneous activity in presence of Folimycin. For this purpose, we stained neurons with DOPE-cypher5E and pre-incubated them at 37°C in presence of TTX and zero external calcium to block AP generation. Additionally, we added Folimycin to convert the fraction of spontaneously recycling SVs into a non-NH_4_Cl-sensitive state. Subsequently, we probed after TTX washout and addition of calcium the apparent size of the recycling pool by stimulation with 900 APs at 20 Hz in presence of Folimycin (**Figure 6A**). If spontaneous activity was drawn from the resting pool, the apparent size of the recycling pool should increase after a period of spontaneous SV turnover in presence of Folimycin. However, we observed an apparent decrease in recycling pool size (**Figures 6B,C**). Thus, the recycling pool could be cross-depleted by preceding spontaneous activity, indicating that SVs are most likely drawn from the recycling pool, as already proposed earlier (Groemer and Klingauf, 2007; Hua et al., 2010).

### SV Are Re-mobilized in a Use-Dependent Fashion

**FIGURE 7:**
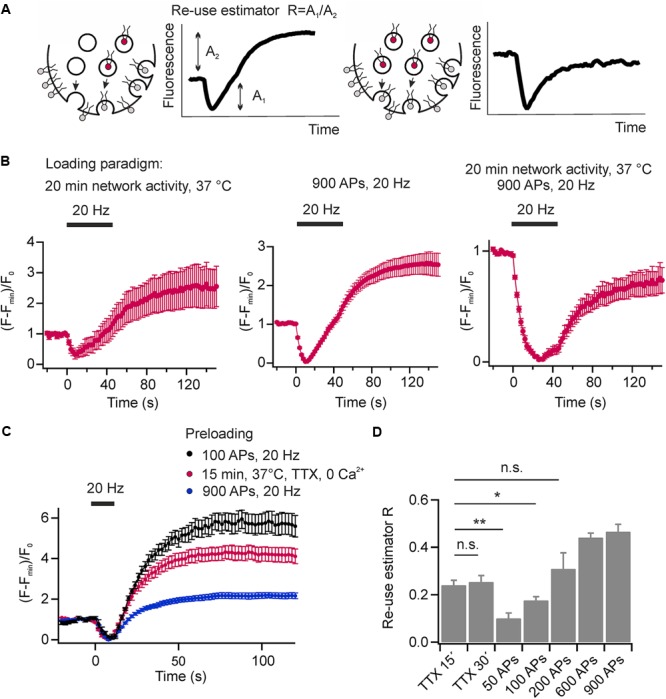
Synaptic vesicles are re-mobilized in a use-dependent fashion. **(A)** Schematic illustration depicting the principle of SV re-use estimation. In scenario 1 (left) not all exocytosed SVs are labeled with DOPE-cypher5E. However, during endocytosis also these SVs will be loaded with DOPE-cyopher5E resulting in an overshoot of fluorescence after endocytosis. In scenario 2 (right), all exocytosed SVs are labeled with DOPE-cypher5E resulting in fully balanced exocytosis/endocytosis amplitudes. We defined the ratio of the apparent exocytosis amplitude A_2_ and the post-stimulus overshoot in fluorescence A_1_ as the re-use estimator R. **(B)** Average normalized fluorescence signal of presynaptic boutons loaded with DOPE-cypher5E by 20 min of network activity at 37°C (left), by a stimulus train of 900 APs at 20 Hz (middle) and by both, 20 min of network activity and 900 APs at 20 Hz (right). Only the latter loading paradigm resulted in fully balanced exocytosis/endocytosis amplitudes upon subsequent mobilization of the recycling pool by 900 APs at 20 Hz (*n* = 5 coverslips, 239 to 474 boutons each). **(C)** Estimation of the degree of SV re-use upon eliciting a test stimulus (200 APs at 20 Hz) after a period of spontaneous loading (15 min, 37°C, TTX, zero external calcium red), or after preloading with 100 APs at 20 Hz (black) or 900 APs at 20 Hz (blue; *n* = 6 coverslips; 87 to 299 boutons each). **(D)** Re-use estimator R for different preloading paradigms as shown in **(C)** indicate preferential re-use of SVs during the test stimulus that had recycled spontaneously compared to those that recycled during evoked activity (^∗∗^*p* < 0.002, ^∗^*p* < 0.04; n.s., not significant, *p* > 0.3, unpaired *t*-test). All error bars represent SEM.

The re-use of SVs is classically analyzed using pHluorin based reporters and pharmacological inhibition of the v-ATPase by Folimycin to convert pHluorin residing in endocytosed SVs into a permanently fluorescent state (Li et al., 2005). However, cypher5E-labeled phospholipids are exogenous tracers that allow visualizing multiple rounds of SV turnover, they offer the unique feature to easily probe for SV re-use after several rounds of activity without pharmacological interference. If only a fraction of exocytosed SVs is labeled with DOPE-cypher5E, fluorescence transients will exhibit an overshoot in fluorescence at the end as also non-labeled exocytosed SVs will be loaded with DOPE-cypher5E during endocytosis (**Figure 7A**, left). If all exocytosed SVs are labeled with DOPE-cypher5E, the amplitudes of exo- and endocytosis are fully balanced (**Figure 7A**, right). Therefore, we can define the ratio of apparent exocytosis amplitude A_2_ over amplitude of fluorescence overshoot A_1_ as a relative SV re-use estimator.

The experiments shown in **Figures 2B, 4B,C** were conducted after maximally loading SVs with cypher5E-labeled lipids by 20 min of network activity, followed by 900 APs at 20 Hz. Using this loading paradigm, we observed fully balanced exo- and endocytosis amplitudes. However, none of these loading paradigms alone is sufficient to obtain equal amplitudes of exo- and endocytosis. In both cases, preloading only by 20 min network activity at 37°C (**Figure 7B**, left) or preloading only by a stimulus train of 900 APs at 20 Hz, (**Figure 7B**, middle) we observed a fluorescence overshoot upon subsequent mobilization of the recycling pool by a test stimulus of 900 APs at 20 Hz. Only a combination of both resulted in balanced exo- and endocytosis amplitudes. (**Figure 7B**, right). This result again indicates that a fraction of SVs, which had not been mobilized during preloading, was mobilized during the test stimulus, i.e., presynaptic boutons feature a rapid mixing of the recycling and resting pools of SVs.

We went on to analyze the fate of SVs retrieved after spontaneous exocytosis in comparison to those retrieved upon evoked activity. After plasma membrane staining, SVs were loaded with DOPE-cypHer5E either by spontaneous activity (1 μM TTX, zero external calcium, 15 or 30 min 37°C) or by electrical stimulation (50–900 APs, 20 Hz). Subsequently, we challenged neurons by a test stimulus (200 APs at 20 Hz, delayed by 60 s relative to the loading stimulus) and estimated the degree of SV re-use by the apparent ratio of exo- and endocytosis amplitudes (**Figure 7C**). About 20% of SVs that had recycled during 15 min of spontaneous activity are re-released upon subsequent evoked activity (**Figure 7D**). Prolonging the TTX incubation period does not lead to a significant decrease of the fluorescence overshoot suggesting that SVs recycle locally and have a high chance to re-occupy the RRP during low frequency activity [spontaneous fusion occurs at a frequency of about one event per 90 s (Murthy and Stevens, 1999)]. In contrast, we observed a significantly smaller ratio of exo-endocytosis amplitudes, i.e., a lower degree of SV re-use, after pre-stimulation with 50 APs at 20 Hz [a stimulation paradigm depleting the RRP and resulting in comparable SV turnover compared to 15 min of spontaneous activity (Sara et al., 2005)]. This finding indicates that SVs recycling during spontaneous activity are preferentially sorted into the RRP of SVs and therefore display a high re-release probability on stimulation. We obtained similar results with DMPE-cypHer5E as fluorescent tracer (data not shown). We further increased the number of APs during the loading phase from 100 to 900 APs. It was not before 200 APs that we observed a similar degree of SV re-use compared to loading by 15 min spontaneous activity. Notably, even after 900 APs, a stimulus depleting all releasable SVs, we found a significant overshoot of the test pulse response, proving fast mobilization from the apparent resting pool during the 60 s interval.

## Discussion

Here, we developed a new class of exogenous probes to monitor SV exo-endocytosis and lipid sorting during SV recycling in general and to analyze SV pool dynamics and SV re-use in particular. These probes based on a pH-sensitive organic dye coupled to phospholipids combine the appreciated features of FM styryl dyes and pHluorin fusion proteins: like FM dyes they do not rely on genetic modification ruling out artifacts due to overexpression (Opazo et al., 2010). Yet unlike FM dyes and like pHluorins they allow monitoring exo-endocytosis repeatedly at the same presynaptic sites. Furthermore with a several 10’s of molecules per vesicle the fluorescence signal variation from vesicle to vesicle is negligible, like for FM dyes (Henkel et al., 1996), but unlike for pHluorin-based probes, where only one or two copies per SV are present (Balaji and Ryan, 2007; Sinha et al., 2011). This is an important feature considering potential analysis of single vesicle release. Nevertheless, for ensemble measurements like shown in **Figures 3D,E** the large spread of amplitudes for the exogenous cypher5E-based tracers is mainly determined by the loading inhomogeneity. However, this lipid-based approach does not provide cellular specificity as targeting of the fluorescent tracer to a subset of cells in a preparation is not possible. In this context, pHluorin-based approaches are still the method of choice allowing for protein expression under control of a cell type-specific promoter.

We were able to perform for the first time analysis of lipid recycling in live hippocampal synapses. In contrast to styryl dyes, which are water-soluble amphiphilic molecules, cypHer5E-labeled phospholipids are derivatives of naturally occurring membrane lipids that stably integrate into the outer leaflet of the plasma membrane. Moreover, it has been shown, that the dynamic behavior of phospho- and sphingolipids in the plasma membrane does not change when fluorescently labeled at the head group, i.e., in the water phase (Eggeling et al., 2009).

We found no evidence for an enrichment of DMPE or sphingolipids in budding vesicles. As for both lipids an affinity for raft-like membrane nanodomains has been reported (Schutz et al., 2000; Eggeling et al., 2009), we conclude that the SV membrane has no raft-like character, underlined by the fact that analysis of the lipid composition of purified SVs has revealed a high cholesterol content but no obvious enrichment in sphingolipids (Takamori et al., 2006). In contrast, we observed highest incorporation efficacy for DOPE, an unsaturated phospholipid with no affinity for raft-like nanodomains. This finding can be explained by curvature-induced lipid sorting during formation and scission of SVs. Small vesicles of ∼ 40 nm exhibit high curvature and lipids with an inverted conical shape are more likely to insert into the inner leaflet. It has been reported, that both, DMPE and DOPE insert preferentially into the inner leaflet of small artificial vesicles *in vitro*, but DOPE displaying an even higher preference for highly curved membranes (Kamal et al., 2009).

There is an ongoing debate on the separation of SVs into distinct SV pools defined by distinct release modes. While some studies identified the recycling pool of SVs as a major source of spontaneous release (Groemer and Klingauf, 2007; Hua et al., 2010; Wilhelm et al., 2010), other studies suggest that the so-called resting pool, a fraction of the total SV pool apparently refractory to stimulation, is mobilized during spontaneous activity (Sara et al., 2005; Fredj and Burrone, 2009; Ramirez et al., 2012). Main evidence was derived in almost all cases from experiments utilizing either FM dyes (Sara et al., 2005; Groemer and Klingauf, 2007; Wilhelm et al., 2010) or pHluorin-based probes (Hua et al., 2010; Ramirez et al., 2012) to optically analyze presynaptic activity. However, FM dyes do not allow monitoring exo-endocytosis repeatedly at presynaptic sites. Probes based on pHluorin report protein recycling and sorting of an overexpressed reporter protein. Notably, for some pHluorin-based reporters, like, e.g., vti1a-pHluorin, conflicting interpretations of release kinetics have been published, claiming either an enrichment of vti1a in the readily releasable pool, i.e., the SV fraction displaying highest release probability (Hoopmann et al., 2010), or an enrichment of vti1a in the resting pool, i.e., the SV fraction of lowest release probability (Ramirez et al., 2012; Crawford et al., 2017).

In contrast, cypHer5E-labeled lipids provide a pure readout of membrane turnover during presynaptic activity regardless of protein complement. By analyzing membrane turnover and estimating SV re-use we accumulated significant evidence that SVs retrieved on evoked or spontaneous activity mix rapidly with the overall presynaptic SV population, confirming previous reports (Groemer and Klingauf, 2007; Hua et al., 2010; Wilhelm et al., 2010). We could show that a small but significant fraction of SVs that is reluctant to release during a first round of evoked activity can be exocytosed during further bouts of stimulation. This finding indicates that the apparent resting pool of SVs upon strong stimulation does not constitute a statically segregated SV population that is refractory to evoked release, but is the result of an acute silencing mechanism for SVs. This finding is in line with earlier work showing resting pool mobilization by inhibition of the Cyclin-dependent kinase 5 (Kim and Ryan, 2010) and developmental dynamic fine-tuning of the fraction of reluctantly releasable SVs in cultured hippocampal slices (Rose et al., 2013). Moreover, we could show that SVs recycling spontaneously are mobilized during subsequent stimulation and display an even higher degree of evoked re-mobilization compared to SVs of the RRP, when released during high-frequency stimulation. These results question the existence of two different SV pools, recycling and resting, for evoked and miniature activity. Instead, SVs are sorted into the queue of releasable SVs in a use-dependent, i.e., frequency-dependent fashion, in accordance with earlier observations showing that low frequency activity re-targets SVs preferentially close to the active zone, where they display high release competence (Vanden and Klingauf, 2006). We cannot ultimately exclude a segregation of SVs into distinct functional pools, but at least spontaneously recycling SVs do not constitute a clearly separated SV population.

However, like for all established tracers of SV recycling, cypher5E-labeled lipids do not provide a spatio-temporal resolution, i.e., signal changes of SV clusters within single presynaptic boutons cannot be resolved. Here, the next challenge is the development of fluorophores, which combine both, pH-sensitivity and suitability for live cell high-resolution techniques like, e.g., stimulated emission depletion microscopy.

In summary, we introduced a new class of optical tracer with unique features, allowing for measuring repeatedly SV recycling at single boutons while simultaneously reporting the ratio of newly exocytosed and re-used SVs without genetic perturbation or pharmacological interference.

## Author Contributions

MK and JK designed the experiments. MK conducted the experiments. MK and JK analyzed the data and wrote the manuscript.

## Conflict of Interest Statement

The authors declare that the research was conducted in the absence of any commercial or financial relationships that could be construed as a potential conflict of interest.
