# Supplementary material for: Novel pH-Sensitive Lipid Based Exo-Endocytosis Tracers Reveal Fast Intermixing of Synaptic Vesicle Pools
Source: Front Cell Neurosci. 2018 Feb 2;12:18. doi: 10.3389/fncel.2018.00018 (PMC5801418; doi:10.3389/fncel.2018.00018)
Supplement: Supplementary file 1 [file Data_Sheet_1.docx]

**Novel pH-sensitive lipid based exo-endocytosis tracers reveal fast intermixing of synaptic vesicle pool**

Martin Kahms^1^, Jürgen Klingauf ^1,2,*^

**^1^**Department of Cellular Biophysics, Institute of Medical Physics and Biophysics, University of Muenster, Robert-Koch-Str. 31, 48149 Muenster, Germany

^2^IZKF Muenster and Cluster of Excellence EXC 1003, Cells in Motion, CiM, Muenster, Germany

**^*^ Correspondence:**

Jürgen Klingauf

[klingauf@uni-muenster.de](mailto:klingauf@uni-muenster.de)

# Supplementary Material


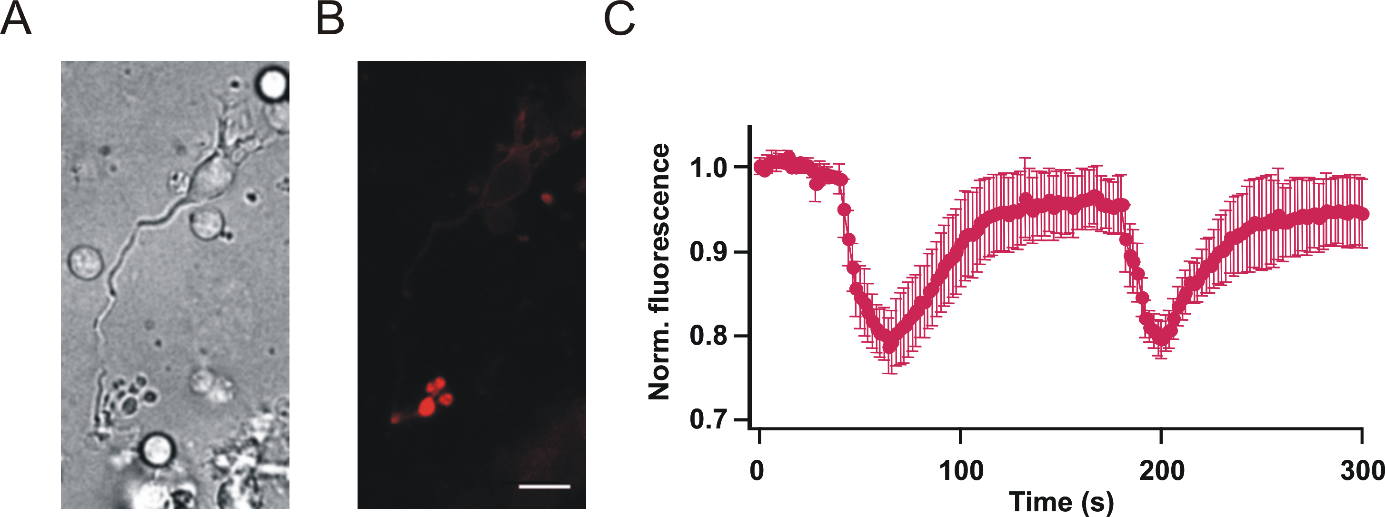


40 mM KCl

40 mM KCl

**Supplementary Figure 1.** **Visualization of exo- and endocytosis in bipolar cells of the retina**. **(A)** Bright field image of an isolated bipolar cell with an intact neurite that forms a branched synaptic terminal. **(B)** Fluorescence image of the same cell after staining with DMPE-cypher5E and depolarization with high potassium solution. Mainly the branched terminal has taken up DMPE-cypher5E during stimulation. Scale bar: 10 µm. **(C)** Average normalized fluorescence signal of DMPE-cypher5E-loaded presynaptic terminals during two further bouts of stimulation with 40 mM KCl solution (n=5). Error bars represent s.e.m.

**
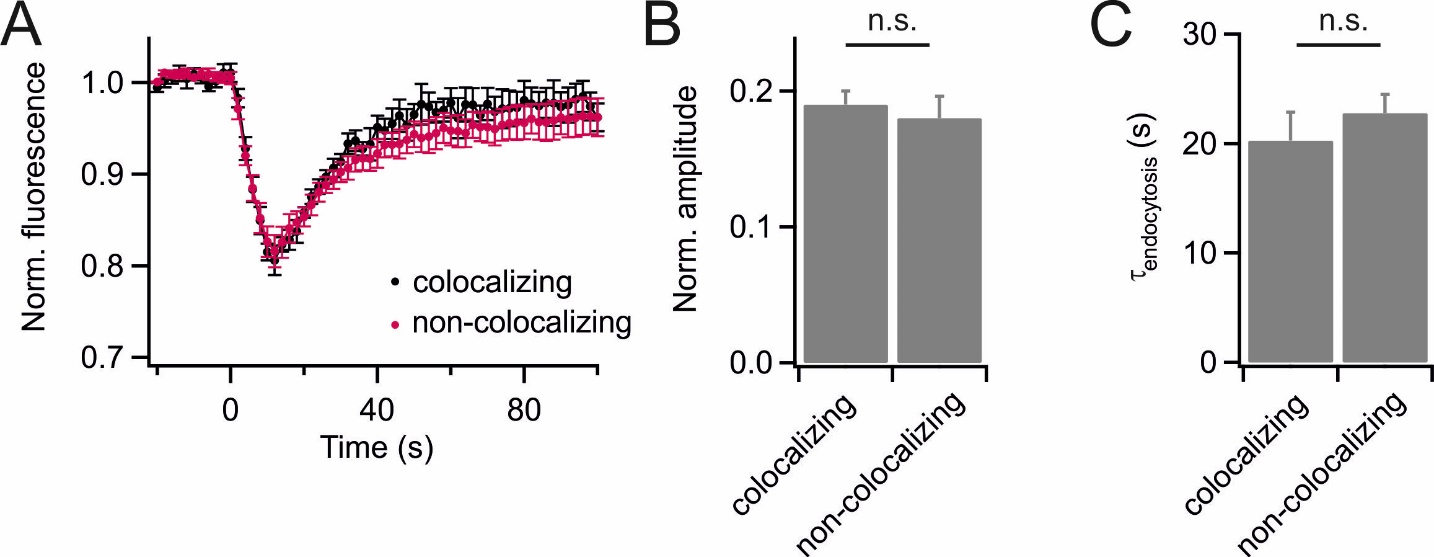
**

**Supplementary Figure 2: Comparison of DMPE-cypher5E signals for boutons expressing Syp1-pHl and boutons devoid of Syp1-pHl fluorescence measured from the same coverslip. (A)** Average fluorescent signals of DMPE-cypher5E in response to 200 APs at 20 Hz for boutons colocalizing with Syp1-pHl (red) and boutons non-colocalizing with Syp1-pHl (black, see Figure 3C). Fluorescent traces were normalized to the NH_4_Cl-responses (n=5 coverslips, 47 to 135 boutons each for boutons colocalizing with Syp1-pHl, 74 to 176 boutons each for non-colocalizing boutons). **(B, C)** Release amplitudes and endocytosis kinetics were similar for colocalizing and non-colocalizing boutons. (n.s.: not significant, p > 0.4, unpaired t-test). Error bars represent s.e.m.

**
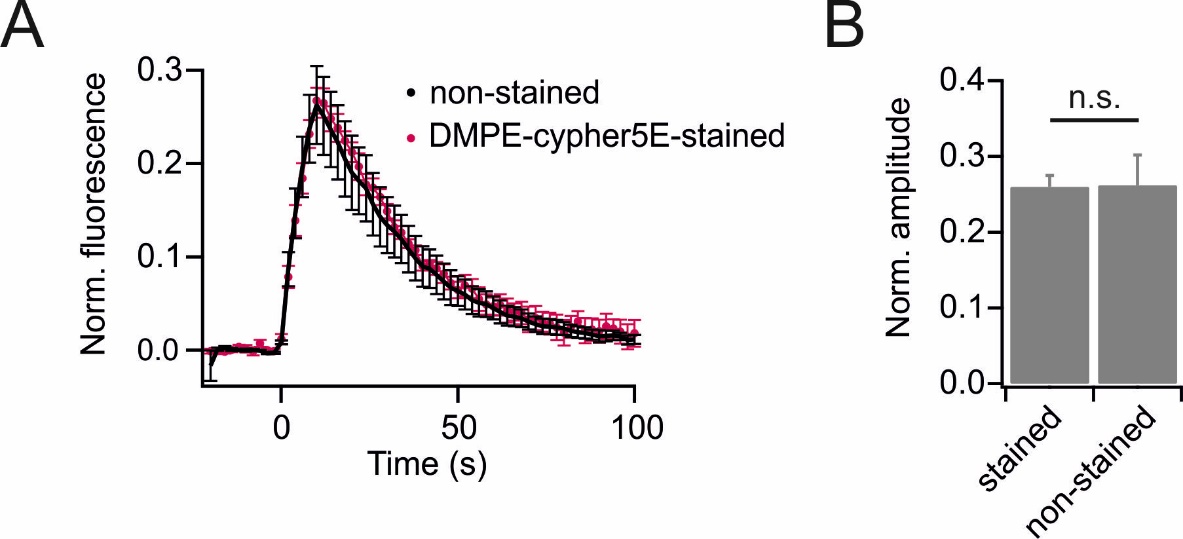
**

**Supplementary Figure 3: DMPE-cypher5E staining does not reduce release probability of SVs. (A)** Average fluorescent signals of Syp1-pHl in response to 200 APs at 20 Hz for DMPE-cypHer5E-stained boutons (see Figure 3D) and non-stained boutons of a different set of coverslips. Fluorescent traces were normalized to the NH_4_Cl-responses (n=5 coverslips, 47 to 135 boutons each for DMPE-cypher5E-stained neurons, 69 to 210 boutons each for non-stained neurons). **(B)** Quantification of release amplitudes revealed no difference in release probability for stained vs. non-stained neurons. (n.s.: not significant, p > 0.5, unpaired t-test). All error bars represent s.e.m.

**
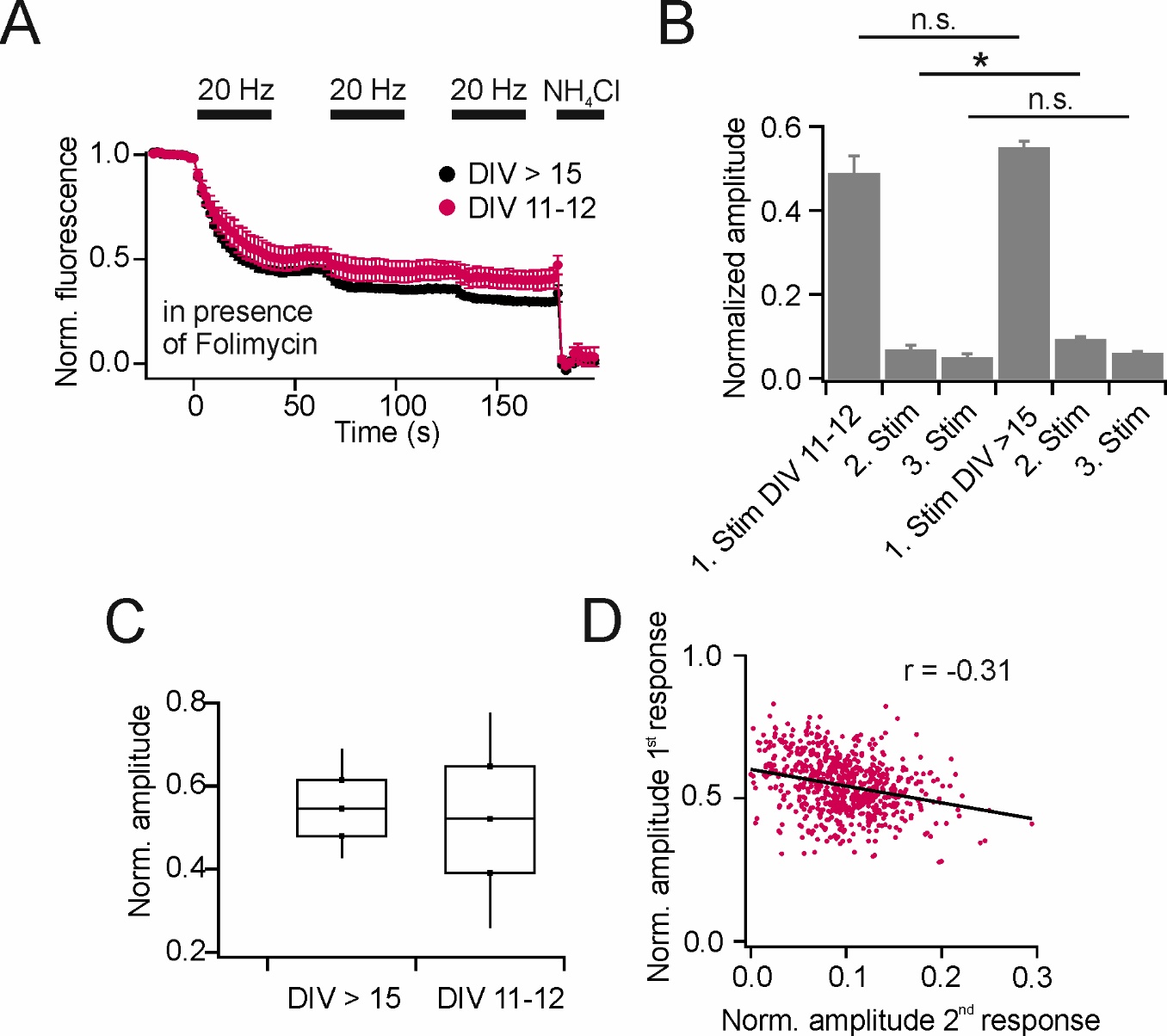
**

**Supplementary Figure 4. Age-dependency of SV pool intermixing and correlation of release amplitudes for subsequent stimuli. (A)** Average normalized cumulative release in presence of 65 nM Folimycin evoked by three 65 s spaced stimulus trains (3x 900 APs at 20 Hz) for neuronal cultures DIV > 15 (black, n=4 coverslips, 110 to 291 boutons each) and neuronal cultures DIV 11-12 (red, n=5 coverslips, 95 to 310 boutons each). **(B)** Quantification of **(A)** revealed only a minor decrease in average release amplitudes for neurons DIV 11-12 compared to neurons at DIV > 15 (* p<0.03, n.s.: not significant, p > 0.3, unpaired t-test). Error bars represent s.e.m. **(C)** Box plot of release amplitudes for individual boutons during the first stimulus train of the experiment shown in **(A)**. Though the average amplitudes are similar, neurons DIV 11-12 display a much broader distribution of release amplitudes compared to neurons DIV > 15 (Boxes represent 25th and 75th percentiles, solid lines the median, and the whiskers represent 10th and 90th percentiles). **(D)** Plot of normalized amplitudes of fluorescent decays of individual boutons for the first stimulus vs. normalized amplitudes of fluorescent decays for the second stimulus of the experiment shown in **(A)** (DIV > 15). The release amplitudes for two consecutive stimulus trains are not correlated (r: Pearson correlation coefficient).

**Supplementary methods**

**Preparation of bipolar cells and analysis of SV recycling.** Rod bipolar cells were isolated from 4-6 weeks old CD1 mice by mechanical trituration after enzymatic digestion. Briefly, animals were euthanized and the retinas rapidly removed and stored in cold, oxygenated, low–calcium buffer (138 mM NaCl, 0.5 mM CaCl_2_ , 0.4 mM MgSO_4_ , 0.5 mM MgCl­_2_ , 5 mM KCl, 0.44 mM KH_2_PO_4_, 0.34 mM Na_2_HPO_4_, 10 mM glucose, 10 mM HEPES, adjusted to pH 7.2). Each retina was cut into small pieces and incubated for 15 min at room temperature in low-calcium buffer containing 2.7 mM L-cysteine (Sigma) and 30 U/ml papain (Fluka). After being washed in low-calcium buffer, pieces were mechanically triturated with a fire-polished Pasteur pipettes and the resulting cell suspension was plated onto clean glass coverslips. Rod bipolar cells were stored in high-calcium buffer (153 mM NaCl, 2.5 mM CaCl_2_, 1 mM MgCl­_2_ , 2.5 mM KCl, 10 mM glucose, 10 mM HEPES, adjusted to pH 7.4) at <10°C for a maximum of 4 h prior to measurements. Bipolar cells were incubated for 15 min with 1 µM DMPE-cypher5E on ice and after lipid washout stimulated with a high-potassium buffer (110 mM NaCl, 2.5 mM CaCl_2_, 1 mM MgCl­_2_ , 40 mM KCl, 10 mM glucose, 10 mM HEPES, adjusted to pH 7.4) for 3 min. After removing the high-potassium buffer, live cell recording was performed applying high-potassium pulses for stimulation of SV release.
